# Impact of Waterpipe Tobacco Pack Health Warnings on Waterpipe Smoking Attitudes: A Qualitative Analysis among Regular Users in London

**DOI:** 10.1155/2015/745865

**Published:** 2015-07-26

**Authors:** Mohammed Jawad, Ali Bakir, Mohammed Ali, Aimee Grant

**Affiliations:** ^1^Department of Primary Care and Public Health, Imperial College London, Hammersmith W6 8RP, UK; ^2^Academic Unit of Primary Care and Population Sciences, University of Southampton, Southampton, Hampshire SO16 6YD, UK; ^3^School of Medicine and Dentistry, Barts and the London, Whitechapel E1 2AD, UK; ^4^Institute of Primary Care and Public Health, School of Medicine, Cardiff University, Neuadd Meirionnydd, Heath Park, Cardiff CF14 4YS, UK

## Abstract

*Background*. Despite the rise in prevalence of waterpipe tobacco smoking, it has received little legislative enforcement from governing bodies, especially in the area of health warning labels. *Methods*. Twenty regular waterpipe tobacco smokers from London took part in five focus groups discussing the impact of waterpipe tobacco pack health warnings on their attitudes towards waterpipe smoking. We presented them with existing and mock waterpipe tobacco products, designed to be compliant with current and future UK/EU legislation. Data were analysed using thematic analysis. *Results*. Participants felt packs were less attractive and health warnings were more impactful as health warnings increased in size and packaging became less branded. However, participants highlighted their lack of exposure to waterpipe tobacco pack health warnings due to the inherent nature of waterpipe smoking, that is, smoking in a café with the apparatus already prepacked by staff. Health warnings at the point of consumption had more reported impact than health warnings at the point of sale. *Conclusions*. Waterpipe tobacco pack health warnings are likely to be effective if compliant with existing laws and exposed to end-users. Legislations should be reviewed to extend health warning labels to waterpipe accessories, particularly the apparatus, and to waterpipe-serving premises.

## 1. Introduction

Waterpipe tobacco smoking is a growing public health concern. It is the predominant tobacco product used by young people in Jordan [1] and Lebanon [2], and a noticeable prevalence is also noted in Western settings, especially among young adults [3]. For example, 1% of adults in Great Britain are regular waterpipe tobacco smokers, a figure which is 10-fold higher among young adults of South Asian origin [4]. In the US, reports from a national survey estimate that 6.1% of adults aged 18–24 years are current waterpipe tobacco smokers [5] and an analysis of the Global Adult Tobacco Survey suggests waterpipe tobacco smoking is increasing across continents with important country-level differences in patterns of use [6].

Among the main motives for initiation and maintenance of use is the notion that waterpipe tobacco smoking is a less harmful alternative to cigarette smoking [7]. Evidence continues to show the contrary; waterpipe tobacco smoke contains many of the chemicals found in cigarettes that are known to induce disease and dependence, such as tar, nicotine, carbon monoxide, and tobacco-specific nitrosamines [8, 9]. A recent systematic review and meta-analysis of the health effects of waterpipe tobacco smoking identified a host of conditions associated with its use, including lung cancer, respiratory disease, low birth weight, and periodontal disease [10, 11]. Despite research efforts to identify waterpipe tobacco smoking as a public health issue warranting attention, interventions promoting its cessation are few [12, 13].

Legislation has played an instrumental role in the decline of cigarette smoking, yet it seems almost absent with regard to waterpipe tobacco use. For example, some cities in the US have smoke-free laws for cigarettes, but waterpipe tobacco is often exempt [14]. Exemptions for waterpipe tobacco are also seen in upcoming European bans on flavoured tobacco which are exclusive to cigarettes and hand-rolled tobacco [15]. This lack of direct legislative attention may have resulted in the proliferation of a largely unregulated industry; London alone has approximately 400 known waterpipe-serving premises [16], the industry has been shown to market their products with misleading descriptors [17]. In the US, waterpipe-serving premises in Oregon exploited a loophole in the Indoor Clean Air Act which exempted retail stores that offered “sampling” to consumers onsite, allowing them to continue serving waterpipe indoors [18].

Placing health warning labels on cigarette packs is a well-established measure to raise awareness of the harms of smoking, promote cessation among continuing users, and prevent initiation among nonusers [19]. Evidence from Lebanon suggests widespread noncompliance of health warning labels on waterpipe tobacco products [20]. One qualitative study in the UK and Canada suggested that the lack of health warnings on waterpipe tobacco packs implied tacit approval of its safety, and where health warnings were salient they were frequently not understood as they were written in Arabic [21]. However, no studies to our knowledge have tested the effectiveness of health warnings on waterpipe tobacco packs; this study sought to address this deficit in the literature.

## 2. Materials and Methods

### 2.1. Design, Sample, and Setting

In order to generate insights from this hard to reach group, focus groups were utilised as a data collection tool over individual qualitative interviews [22]. Moreover, we restricted the size of the focus groups in order to enable a high level of researcher control [23]. Our sample frame consisted of London university students, a group known to have particularly high prevalence of waterpipe tobacco smoking [4]. In 2014, two researchers (Ali Bakir and Mohammed Ali) acted as gatekeepers to recruit their peers who were regular waterpipe tobacco smokers adopting snowball sampling to recruit additional participants [24], a technique used previously to target this group in London [25]. Participants were recruited in person (face-to-face) and by electronic media (emails, phone calls, and social media). They were invited to attend focus group discussions to discuss attitudes towards waterpipe tobacco pack health warnings. A topic guide was developed using a framework based on the theory of reasoned behavior [26].

### 2.2. Waterpipe Tobacco Pack Health Warnings

**Figure 1:**
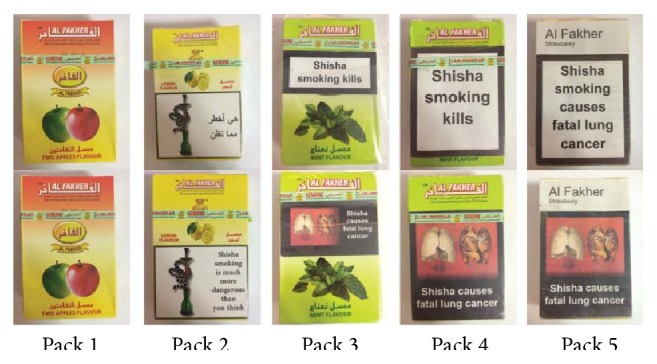
Health warning labels (top row = front of pack; bottom row = back of pack).

Existing waterpipe tobacco packs are known to be noncompliant with tobacco health warning requirements [20]. In the UK, one of the most known waterpipe tobacco brands is “Al-Fakher” and two different packs of these were purchased at a local retail store in London. One pack contained no visible health warning labels on its front or back surface (Figure 1, Pack 1) and one contained health warning labels that were noncompliant with existing legislation as it contained bilingual (English/Arabic) health warnings (Figure 1, Pack 2). We purchased two more Al-Fakher waterpipe tobacco packs with no visible health warnings and used these to apply our own health warnings. For one pack we created health warnings to comply exactly with current English legislative requirements on tobacco health warnings [27, 28], which involved a text warning covering the front 30% of the pack, and a graphic plus text warning covering the back 40% of the pack (Figure 1, Pack 3). This is also compliant with requirements of the European Union Tobacco Products Directive [29]. For the other pack we created health warnings to comply with recommendations based on the Hammond review of health warnings labels worldwide [19] (which comply with potentially future EU legislation), which involved a text warning covering the front 75% of the pack, and a graphic plus text warning covering the back 75% of the pack (Figure 1, Pack 4). Finally, we created a waterpipe tobacco pack to mimic standardised packaging (“plain packs,” Figure 1, Pack 5), based on the legislative guidelines outlined by Australia [30]. Packs 3–5 contained the same health warnings (front: “Shisha smoking kills”; back: “Shisha causes fatal lung cancer”), based on the best available evidence on waterpipe health outcomes [10], which we stuck underneath the cellophane of the tobacco pack to promote a sense of authenticity. In order to reduce response bias, we did not tell focus group participants that some waterpipe tobacco pack health warnings were created by us.

### 2.3. Pilot Focus Group

We conducted a pilot focus group among three participants (Group 1). This confirmed the authenticity of our health warnings and enabled the possibility of feedback to the facilitators (Ali Bakir and Mohammed Ali) from a more experienced qualitative researcher (Aimee Grant). We provided a range of health warning messages to this group (“Using coal to heat shisha tobacco causes carbon monoxide poisoning,” “Shisha smoking causes fatal cancers,” “Shisha can spread tuberculosis,” “Shisha smoking can cause premature death,” “Shisha smoking harms pregnancy,” “Shisha smoking kills,” and “The water in shisha does not filter harmful chemicals”) and participants indicated that shorter adverse health outcomes were better received than longer ones. The two health warnings “Shisha smoking kills” and “Shisha causes fatal lung cancer” were selected as examples of better received health warnings, which we incorporated across both packs we created. We also tested the appropriateness of our prompts and questions after reading the transcript and hearing its audio recording. Suggestions for improvement were fed back to the facilitators via a series of meetings with the research team.

### 2.4. Data Collection and Analysis

Twenty participants took part in five focus groups with a mean of four participants per group (range 3–6). Focus groups occurred in university campus meeting rooms (Groups 2 and 3) or at one of the participants' homes during a meeting of friends which invariably involved waterpipe tobacco smoking (Groups 1, 4, and 5). No discernable difference occured in participant engagement or focus group duration which could be attributed to the research venue. We provided all participants with written information about the study and informed consent was obtained prior to focus group discussions. All focus groups were conducted by Ali Bakir and Mohammed Ali, who alternated between roles as facilitator and note-taker in each focus group, providing feedback to one another in an iterative process. All focus groups were audio-recorded and transcribed verbatim. Mean focus group length was 39 minutes. Thematic analysis [31] was undertaken by one researcher (Aimee Grant), facilitated by the use of NVivo 10. 20% of the data was independently coded by a second researcher (Mohammed Ali), and a high proportion of interrater reliability was obtained, with minor inconsistencies discussed and resolved. This study was approved by the Imperial College Research Ethics Committee.

## 3. Results

### 3.1. Participant Characteristics

The mean age was 24.4 ± 3.2 years and 17 of the 20 participants were male. Half were Arab, nine were of South Asian ethnicity, and one participant was White British. Twelve were students, seven were employed, and one was self-employed. Twelve only smoked waterpipe, and eight smoked both waterpipe and cigarettes. Mean age of waterpipe initiation was 17.9 ± 2.8 years (range 13–25 years). Eight smoked waterpipe less than weekly, six smoked weekly, three smoked two to three times per week, and three smoked daily. Only two participants had made previous quit attempts.

### 3.2. Themes

Our findings are divided into the impact of health warnings on perceived attractiveness of waterpipe smoking, the clarity of health warnings on the five waterpipe tobacco packs, the perceived impact on compliant health warning labels, and participants' real world exposure to health warning labels. Throughout, the impact of standardised (plain) packaging and the impact on nonusers will be discussed.

#### 3.2.1. Health Warnings and Attractiveness

Overall, participants found packages without health warnings (Pack 1) or with UK compliant health warnings (Pack 3) most attractive. Attractiveness decreased as the size of health warnings increased (Pack 4), or as the packaging lost its branding (Pack 5):

[Pack 1] may taste nicer also because if it does not have a health warning on it, you would assume that it would taste nicer, coz something with a health warning on it you would assume it has chemicals in it so it wouldn't taste that nice…. (Group 5, Participant 1: male, aged 23, Indian ethnicity, weekly waterpipe-only user)

I think they ruined the look of the mo'assal (tobacco) [on Pack 5]. There is no brand name, it covers the whole thing with the [health warning] picture. (Group 4, Participant 3: male, aged 29, Arab ethnicity, twice weekly waterpipe user, dual waterpipe/cigarette user)

In every focus group, the concept of the colourfulness of packaging was discussed, and references to the waterpipe tobacco packaging (Packs 1–4) looking like “candy” were frequent. Alongside this, many of the participants stated that the packaging would be attractive to children. The appeal of brightly coloured packaging for adults, however, was contested. Some participants thought that the colourful waterpipe tobacco packages (Packs 1–4) were generally unsophisticated and unattractive. One participant noted that it would be embarrassing to be seen with such a package:

[Pack 1 is] too loud and they look a bit messy…like the other one [Pack 2] is like bright yellow thing, I don't really want to be carrying that around… [Pack 2] could be like candy or lollipops…. (Group 1, Participant 1: male, aged 22, White British ethnicity, weekly waterpipe-only user)

Other participants suggested that the colour was attractive and that by making the packaging “plain” or standardised (Pack 5) the product would be less attractive to them.

#### 3.2.2. Clarity of Health Warnings

The existing, noncompliant warning “Shisha smoking is more dangerous than you think,” accompanied by a picture of a snake wrapped around a waterpipe (Pack 2), was viewed as less clear than “Shisha smoking kills” alongside a UK compliant pictorial health warning (Packs 3–5). Speaking about Pack 2,

It's not really a warning, you look at the picture more than the words really and the picture is of a shisha so it's not very intimidating. (Group 4, Participant 3: male, aged 29, Arab ethnicity, twice weekly waterpipe user, dual waterpipe/cigarette user)

In addition to this, bilingual (English/Arabic) health warnings found on Pack 2 were viewed as a distraction, particularly for users who did not understand Arabic. For some participants, making packaging looking more similar to cigarette packaging (Packs 3–5) reinforced that waterpipe smoking was dangerous for health: “I think that one (Pack 3) is more like cigarette box and we associate cigarettes as being bad….” (Group 4, Participant 5: male, aged 26, Arab ethnicity, 3x week waterpipe user, dual waterpipe/cigarette user).

#### 3.2.3. Perceived Impact of Health Warnings on Waterpipe Smoking

Participants varied in the extent to which they reported that warning labels would influence their waterpipe smoking behaviour. Some participants noted that even regular waterpipe users would not choose to buy waterpipe tobacco in plain packaging (pack 5), but throughout the focus groups, participants noted the importance of addiction on behaviour:

Interviewer: Would you be deterred by messages, by packages like these [Packs 3 and 4]?

Participant: Slightly yeah, it's hard, if you are in the habit of smoking it's hard for you to keep off easily, but it does have some sort of impact. (Group 4, Participant 5: male, aged 26, Arab ethnicity, 3x week waterpipe user, dual waterpipe/cigarette user)

For those who reported that their behaviour would not be affected, viewing health warnings was still perceived to be a negative experience:

[if health warnings were on waterpipe tobacco packaging] it will be annoying and it won't have an effect on me, that's for sure… It'll be like I wasn't to see the packaging, I don't want to see that… It wouldn't make me any less of thinking about quitting… I will quit everything in my life before I quit shisha…shisha is like my blood…. (Group 2, Participant 2: male, aged 23, Indian ethnicity, daily waterpipe user, dual waterpipe/cigarette user)

Participants reported that they felt that making waterpipe tobacco less attractive, by introducing standardised packaging (Pack 5) or large health warnings (Pack 4), would reduce the appeal for young people. However, the lack of exposure to packaging was noted as a disadvantage for attempting to expose young people to health warnings.

#### 3.2.4. Exposure to Waterpipe Tobacco Packaging

There was wide agreement from participants that they did not regularly come into contact with waterpipe tobacco packaging. Three reasons were provided for this. Firstly most participants used waterpipes in public venues, where the pipe was prepared by venue staff and presented prepacked with tobacco. Secondly, where participants shared waterpipes with friends another person would be involved in preparing the pipe. Thirdly, participants purchased waterpipe tobacco that was sold (illegally) in plastic bags or unbranded containers or from other countries:

I don't think many people have shisha in their houses. They smoke shisha at the cafes, and at cafes we don't see the package at all… I never ever in my life…bought from a British [shop]…. (Group 2, Participant 6: male, aged 23, Arab ethnicity, weekly waterpipe user, dual waterpipe/cigarette user)

However, six participants noted that when they smoked waterpipe in public venues, a health warning was attached to the pipe. Participants stated that they found viewing these warnings uncomfortable (“annoying”) whilst others actively attempted to avoid the warning:

When I have a shisha, I turn it around; I don't like looking at it…it is putting me off… So I turn the pictures around…the text doesn't bother me…whereas the pictures it will automatically register regardless of whether I consciously look at it or not. (Group 1, Participant 2: male, aged 23, Pakistani ethnicity, weekly waterpipe-only user)

One participant reported that the presence of health warnings attached to the pipe deterred him from smoking waterpipe in that café.

## 4. Discussion

To our knowledge this study is the first to assess the impact of waterpipe tobacco pack health warning labels on waterpipe tobacco users. Using a legislative gradient of health warnings ranging from noncompliant (Pack 1) to standardised (“plain”) packs (Pack 5), participants' reactions to them were “dose-responsive”; that is, the bigger the health warning/more plain the packaging, the greater the negative response. This was especially true regarding package attractiveness and the perceived impact of health warnings on waterpipe smoking.

Health warning labels on existing UK waterpipe tobacco packs appear ineffective and participants suggested their absence and subsequent colourful packaging may appeal to children. Indeed, young people and adolescents appear particularly vulnerable to waterpipe tobacco smoking in both Western and Middle Eastern settings [1, 2]. Irrespective of the health warning compliance level of waterpipe tobacco packs, the lack of exposure appeared to highlight a fundamental flaw in existing tobacco control legislation; namely, waterpipe tobacco smoking is not homogenous with cigarette smoking. While health warning labels are apparent to cigarette users at point of sale and the point of consumption, opportunities to present health warnings to waterpipe users appear concentrated at the point of consumption. Some local governments in the UK appear to be attaching health warnings to waterpipes in shisha cafes, and our findings show some evidence to suggest that this may be an effective way of communicating health risks to waterpipe users.

The literature is bereft of information on health warning labels. In a qualitative study among English and Canadian waterpipe smokers, some recalled seeing health warnings in foreign languages only, or none at all [21]. A study in Lebanon showed that the majority of the 74 purchased waterpipe tobacco products contained text-only warnings covering an average of 3.5% of their total surface area [20]. On waterpipe tobacco retail websites, only 4% contained a health warning of any description on any page [32]. In a qualitative study of staff responsible for enforcing laws on waterpipe-serving premises, several provided health warning lanyards to premises for placement over the waterpipe apparatus; however most premises were noncompliant [16].

This study preliminarily demonstrates the benefits of increasing health warning label size and moving towards standardised (“plain”) waterpipe tobacco packs. However due to lack of exposure, emphasis should be placed on communicating health risk on waterpipe apparatuses and other accessories, particularly at waterpipe-serving premises. Consideration should be given to displaying health warning posters on premises dedicated to the sale of waterpipe tobacco. Better control of waterpipe tobacco sales is needed in the context monitoring the illicit market, which appears to contribute a significant proportion of waterpipe tobacco sales in the UK [16]. Communicating health risks is somewhat fraught with inconsistent and potentially harmful messages from public health staff and organisations [33], and efforts should be made to communicate a unified and clear message of harm. To our knowledge only Lebanon has laws on specific messages for waterpipe tobacco pack health warning labels [34], which could be used as a basis for other countries looking to implement waterpipe-specific health messages.

This is the first published study to evaluate the impact of waterpipe tobacco pack health warnings on waterpipe smoking behaviour. Health warning labels were created in accordance with existing and (potential) future European legislation and were piloted for authenticity. However this sample relied on a small, convenience sample limited to one area of the UK. We also did not seek to select a representative sample of existing waterpipe tobacco products on the UK market and limited our study to one brand. Furthermore, we only tested one set of health warnings (“Shisha smoking kills” and “Shisha causes fatal lung cancers”) and we anticipate a slightly different response to messages communicating the benefits of cessation or referral to a cessation service.

## 5. Conclusions

Waterpipe tobacco pack health warning labels are likely to be more effective if larger or if displayed as part of a standardised (“plain”) pack. However, due to the inherent ways in which it is smoked, waterpipe tobacco pack health warning labels may have limited exposure to waterpipe users, especially in a café setting. Laws on waterpipe tobacco pack health warning labels should be revised to accommodate for this difference by extending health warnings to waterpipe accessories and parts of waterpipe-serving premises.
